# Common auricular acupoint patterns for postoperative recovery across laparoscopic surgeries: a data mining-based systematic analysis

**DOI:** 10.3389/fmed.2026.1708106

**Published:** 2026-03-17

**Authors:** Guozhi Liu, Wenxin Wu, Guanglin Sang, Jiuli Li, Chang Liu, Yingqi She, Yixuan Ou, Huibing Chen

**Affiliations:** 1 Department of Nursing, Zhongshan Hospital of Traditional Chinese Medicine Affiliated to Guangzhou University of Traditional Chinese Medicine, Zhongshan, China; 2 Proctology Department, Zhongshan Hospital of Traditional Chinese Medicine Affiliated to Guangzhou University of Traditional Chinese Medicine, Zhongshan, China; 3 Intensive Care Unit, Zhongshan Hospital of Traditional Chinese Medicine Affiliated to Guangzhou University of Traditional Chinese Medicine, Zhongshan, China

**Keywords:** auricular therapy, data mining, gastrointestinal dysfunction, selection rule, traditional Chinese medicine

## Abstract

**Background:**

Gastrointestinal dysfunction is a common complication of patients following laparoscopic surgery, prolonging recovery, and increasing healthcare costs. With Western medicine approaches having limitations, traditional Chinese medicine (TCM), particularly auricular therapy, has shown promise in managing this condition, previous research has lacked standardization in acupoint selection and has not fully utilized data mining techniques to uncover patterns in acupoint combinations. This study aims to conduct an exploratory analysis and pattern recognition through extensive literature review and data mining, thereby identifying common auricular acupuncture point patterns in laparoscopic surgery.

**Methods:**

This study systematically analyzed auricular therapy for gastrointestinal dysfunction after laparoscopic surgery. Data were retrieved from multiple Chinese and English databases up to April 30, 2025, using relevant search terms. Studies were filtered based on inclusion criteria and exclusion criteria. The included studies were standardized according to the Chinese national standard for auricular point nomenclature. Study information was organized using EndNote X9 and Excel 2021. Data underwent frequency analysis, association rule analysis, cluster analysis and network analysis to uncover patterns in acupoint selection and combinations.

**Results:**

A total of 118 papers were included in the study, featuring 28 types of auricular points used 632 times. High-frequency points included Stomach, Large Intestine, and Small Intestine. Association rule analysis revealed 48 rules with lift values greater than 1, indicating significant correlations between certain acupoint combinations. Cluster analysis grouped the 28 auricular points into five clusters, while network analysis identified 12 strong connections between acupoints.

**Conclusion:**

This study provides a systematic analysis of auricular acupoint selection patterns for gastrointestinal dysfunction after laparoscopic surgery, as the Stomach-Large Intestine-Sanjiao acupoints as the foundational protocol, Sympathetic-Shenmen as the synergistic module, and Spleen as the pivotal modulator for pattern differentiation. The findings highlight the potential of auricular therapy in this context and offer a reference for clinical practice. Future studies should further validate these type-specific patterns by expanding multi-center data sampling and integrating modern medical diagnostic techniques to enhance the reliability and applicability of the results.

## 1 Introduction

Laparoscopic surgery, which is a minimally invasive procedure performed through a small incision in the abdominal wall, where laparoscopic instruments and a camera are inserted with the help of real-time image guidance. Compared with traditional open abdominal surgery, its core advantages include less trauma, less bleeding, quicker recovery, and a more aesthetically pleasing incision (1, 2). Since the first laparoscopic cholecystectomy in 1987, this technique has been widely used in the fields of gastrointestinal, hepatobiliary, gynecological and urological surgery, and has become an important symbol of modern surgery.

Gastrointestinal dysfunction after laparoscopic surgery is a common complication that significantly affects patients' postoperative recovery and quality of life. Song et al. (3) reported that the incidence of postoperative gastrointestinal dysfunction ranged from 4.5% to 71.2%, which plagues healthcare professionals and patients, affecting the recovery process of patients and leading to prolonged hospital stays and increased financial burdens (4, 5). This condition falls within the scope of TCM syndromes such as pi-man, abdominal distension, abdominal pain, intestinal obstruction, intestinal knotting, and constipation. Western medicine typically addresses these issues through gastrointestinal decompression, fasting, and the use of gastrointestinal motility drugs. However, these methods have limitations in clinical efficacy, including drug tolerance issues and the potential for intestinal dysbiosis (6).

Traditional Chinese medicine (TCM) has accumulated extensive experience in treating gastrointestinal diseases (7, 8). Auricular therapy, a unique TCM therapy, has demonstrated remarkable efficacy in alleviating postoperative gastrointestinal dysfunction (9). The ear, as a micro-acupuncture system, contains numerous acupoints corresponding to different body parts and organs. Stimulating these acupoints can regulate the functions of the corresponding organs and improve gastrointestinal motility (10, 11). However, existing research exhibits two major limitations: Firstly, stimulation methods varied across studies, including auricular seed therapy, acupoint massage, cupping, acupuncture, and electroacupuncture. Treatment frequency, course duration, and follow-up outcome measures (symptom scales, physiological indicators, functional scores, etc.) also showed significant differences between studies, yet a standardized selection criterion is lacking (9, 12), which manifested in three aspects: core point selection, auxiliary point combinations, and selection rationale (13–15). Secondly, most studies remain at the level of clinical observation, failing to utilize data mining techniques to reveal the intrinsic patterns governing acupoint combinations. The current state, it is unclear whether certain combinations of acupoints are commonly practiced or are the preference of individual researchers, which leads to arbitrary point selection in clinical practice, highlighting an urgent need for systematic analysis of selection patterns to guide standardized application. Our study uses large-scale literature synthesis and data mining methods to identify common patterns in the selection of auricular points across different laparoscopic surgical procedures. By analyzing aggregated clinical report data, we aim to identify patterns observed in practical clinical applications and suggest testable research hypotheses regarding point combinations.

Notably, laparoscopic surgery encompasses diverse subtypes across general surgery, gynecology and urology, with postoperative gastrointestinal symptoms varying significantly by surgical site and type. For example, laparoscopic gastrointestinal surgery is associated with severe gastrointestinal dysmotility due to intraoperative intestinal manipulation, while laparoscopic uterine surgery presents with milder gastrointestinal disturbances but prominent pelvic discomfort. By summarizing the common acupoints and their underlying mechanisms, may provide methodological reference and research directions for future comparative clinical studies.

## 2 Data and methods

### 2.1 Data resource

A systematic search in Chinese databases including China National Knowledge Infrastructure (CNKI), Chinese Scientific Journals Database (CQVIP), China Biology Medicine (CBM), and Wanfang Data, and English databases including PubMed, Embase, and Cochrane Library was conducted to retrieve papers.

The time span for the paper retrieval was set from the foundations of the databases to April 30, 2025, using the following search terms: “Auricular Acupuncture” OR “Ear Acupuncture” OR “Auricular Therapy” OR “Ear Acupressure” OR “Auricular Point” OR “Ear Point Stimulation,” “Post-laparoscopic Surgery” OR “Post-laparoscopy” OR “Laparoscopic Surgery” OR “Laparoscopy” OR “Postoperative Recovery after Laparoscopy,” “Gastrointestinal Dysfunction” OR “GI Dysfunction” OR “Gastrointestinal Motility Disorder” OR “Postoperative Gastrointestinal Recovery” OR “Gastrointestinal Function Recovery” OR “Digestive System Dysfunction” OR “Postoperative Gastrointestinal Recovery.”

### 2.2 Criteria for inclusion and exclusion

#### 2.2.1 Inclusion criteria

Studies were included if: (1) they were about randomized controlled trials and quasi-trials; (2) they were subject to patients who had undergone laparoscopic surgery; (3) they were with auricular therapy as the main intervention, alone or in combination with other treatments; (4) they were clearly given the specific auricular acupoint; (5) the outcome index was the recovery of gastrointestinal function.

#### 2.2.2 Exclusion criteria

Studies were excluded if: (1) they were published in more than one place; (2) they only presented a program or an abstract; (3) their data were incomplete or unobtainable; (4) they were with ambiguous diagnosis and primary treatment; (5) they were about Meta-analysis, systematic review, review, case report or animal experiment.

### 2.3 Standardization

According to the “National Standard of the People's Republic of China: Auricular Point Name and Location” (GB/T 13734-2008) (16), the name and location of auricular points were standardized. For instance, “Cortex” was changed to “Subcortex,” “Pancreas” and “Gallbladder” were changed to “Pancreas-Gallbladder,” “Abdominal” was changed to “Abdomen,” “Brain Point” was changed to “Brain,” etc.

### 2.4 Database establishment

All the retrieved studies were imported into EndNote X9 (https://endnote.com) for screening, deduplication and classification. In addition, Excel 2021 was used for data extraction and database establishment. According to the title, year, author information, auricular point location, and intervention method, the study information was input and sorted out. If there were two or more groups of schemes used alternately in one article, these schemes will be individually segregated and systematically entered into the data sheet. If the primary acupoints in a literature source include multiple groups due to different syndrome differentiation types, the corresponding prescriptions will be divided into distinct entries and recorded separately.

To ensure the authenticity and accuracy of the data, each eligible study was reviewed independently by two researchers, if there was any difference in information input, a third researcher was responsible for proofreading and reviewing to prevent duplications, input errors, and omissions.

### 2.5 Data mining analysis

The frequency method was utilized to count the preparations of prescriptions that met the inclusion and exclusion criteria in the Excel worksheet. Association rules (17, 18) were generated from these frequent item sets, followed by analysis based on the number of the common appearances of items. The analysis results were expressed in terms of “Support,” “Confidence,” and “Lift.” Support is defined as the frequency with which the items in the antecedent and consequent appear together in the dataset. A higher support value indicates that the rule is more general and applicable to a larger portion of the data. Confidence is a reliable metric for evaluating the efficacy of the rule. This probability is defined as the probability that a specific occurrence will transpire subsequent to the occurrence of another specific event. A confidence value greater than 80% suggests that the rule is reasonably reliable. Lift is a method of comparing the observed support of a given rule to what would be expected if the antecedent and consequent were independent. A lift value greater than 1 indicates a positive correlation between the antecedent and the consequent, signifying that the occurrence of the antecedent increases the likelihood of the consequent occurring. Cluster analysis was conducted utilizing the OriginPro 2025, with a genealogical map being developed in order to explore the clustering relationship between the acupoints. Cluster analysis used Euclidean distance to calculate acupoint similarity and Ward's method for cluster merging. This method minimizes within-cluster sum of squares, which is suitable for frequency data clustering in this study and consistent with common practices in similar data mining research (19). Each sample set in the data sets is regarded as a cluster, and then the clusters with close distances are merged step by step to achieve the expected number of clusters. Network construction based on the Apriori algorithm by SPSS Modeler 18.0 was established, and X was set as the first term and Y as the second term. The nodes represent individual acupoints and the lines represent the correlation between acupoints. The thickness of the line is indicative of the strength of the correlation, with a thicker line denoting a stronger correlation. The dotted line, conversely, indicates a weaker correlation. The lower threshold for strong connections (35) and upper threshold for weak connections (15) were determined based on the data distribution: the 75th percentile (P75) of all acupoint co-occurrence counts was 35, and the 25th percentile (P25) was 15. This setting aligns with the threshold logic of network analysis (17), ensuring it is data-driven rather than arbitrary.

From a methodological perspective, this study does not constitute an evidence synthesis guided by effect size estimation. Consequently, network meta-analysis (NMA) is not applicable in this context. The majority of the included studies did not report comparable quantitative outcome measures, and intervention protocols exhibited significant variations in acupoint combinations, stimulation modalities, treatment durations, and outcome definitions. Moreover, the paucity of randomized controlled trials, in conjunction with the heterogeneity of effect measure reporting across trials, hinders the transferability and consistency prerequisites necessary for network meta-analysis. The primary objective of this study is to explore patterns in ear acupuncture point selection within published clinical reports. The application of data mining methodologies has been demonstrated to be effective in the identification of co-occurrence structures and pairing patterns within highly heterogeneous clinical data sets characterized by inconsistent effect sizes. Consequently, this study adopts a pattern-recognition approach rather than an outcome-effect-assessment approach. The objective of the research is to describe patterns of point selection and propose research hypotheses regarding commonly used pairings. The primary focus is on describing these patterns and hypotheses, rather than comparing the efficacy differences between different interventions.

## 3 Results

### 3.1 General information

**Figure 1:**
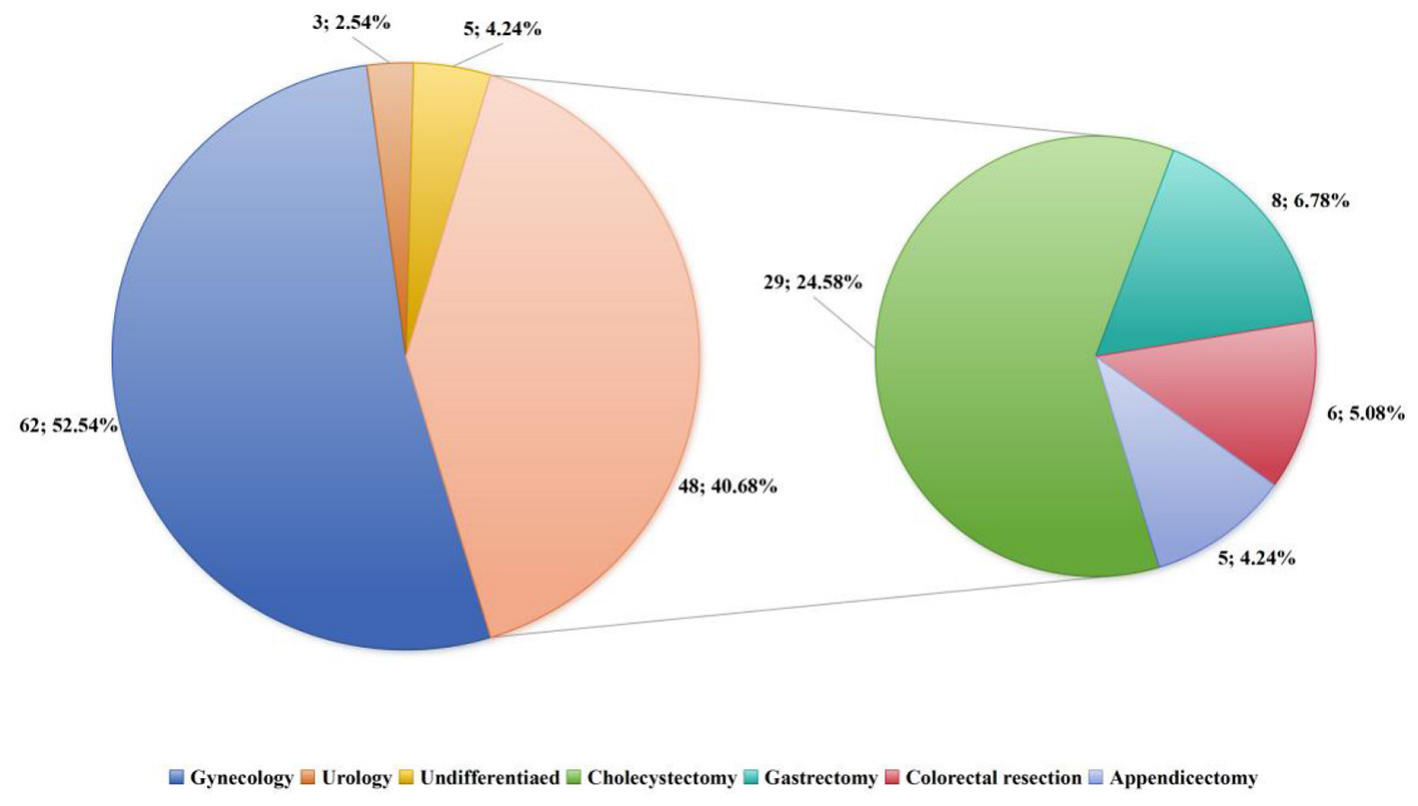
The proportion of different startification of laparoscopic surgeries.

**Figure 2:**
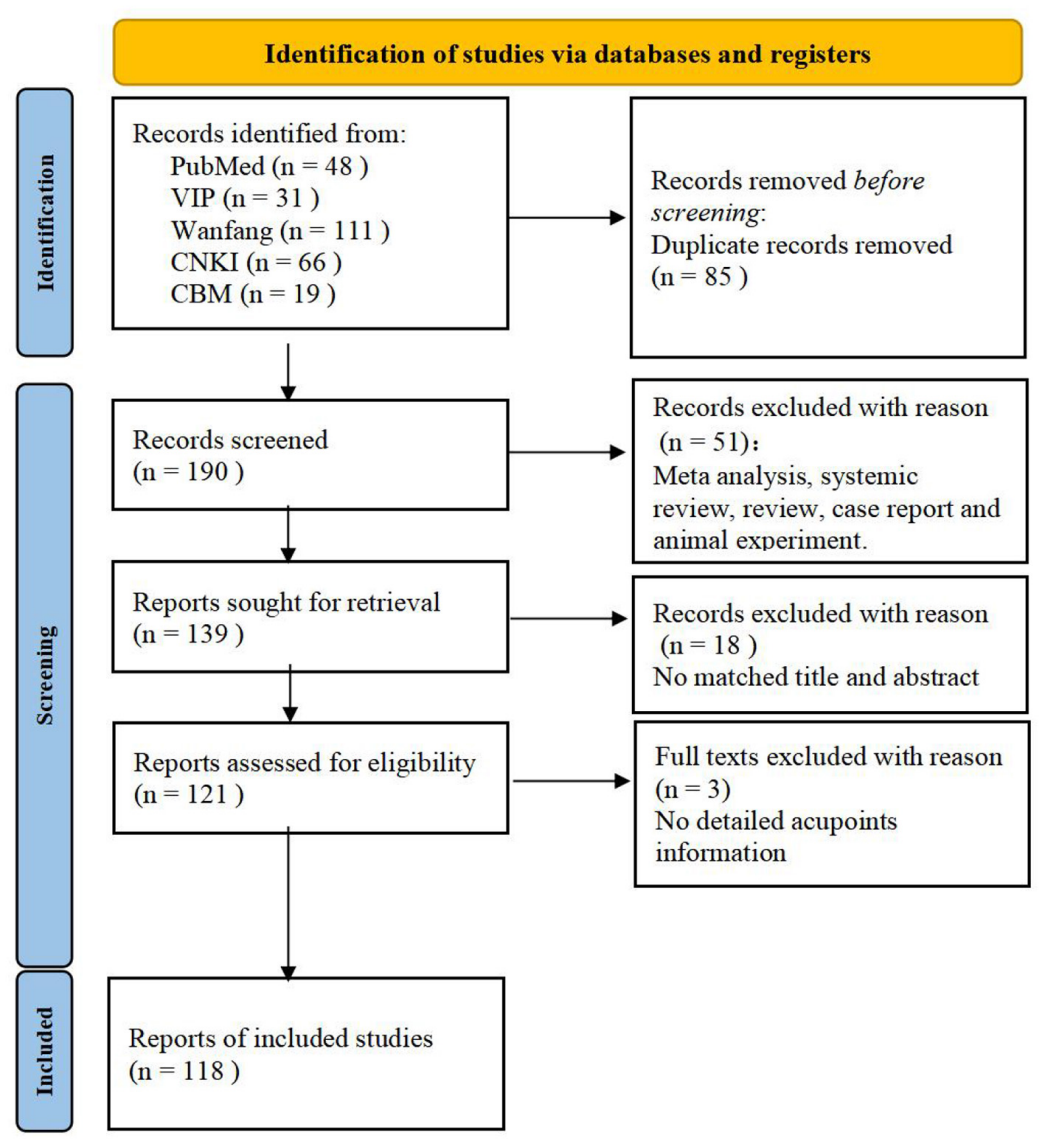
Flow chart of the study design.

A total of 275 papers were retrieved from the databases initially, with 85 remaining after removing duplicates. Then, 69 papers were excluded judging by their titles and abstracts. Meanwhile, 3 papers were excluded with unclear acupoints selection. Finally, 118 papers were included, the proportion of different stratification of laparoscopic surgeries is shown in Figure 1, we categorize laparoscopic surgical sites into gynecology, urology, general surgery, and undifferentiated. Within general surgery, further distinctions are made between cholecystectomy, gastrectomy, colorectal resection, and appendectomy. The Kappa coefficients for literature screening and data extraction by the two researchers were 0.87 and 0.89, respectively (both >0.8), indicating excellent consistency. A total of 8 controversial cases (6.78% of the total 118 studies) required intervention by a third researcher, and final consistency was achieved by cross-checking the original literature. The detailed information can be seen in Figure 2. All included randomized controlled trials (RCTs) were evaluated using the Cochrane Risk of Bias Tool. Among the 89 RCTs, 42 (47.19%) were judged as low risk of bias, 40 (44.94%) as moderate risk, and 7 (7.87%) as high risk. High-risk bias mainly originated from “unclear allocation concealment.”

### 3.2 Frequency analysis

**Figure 3:**
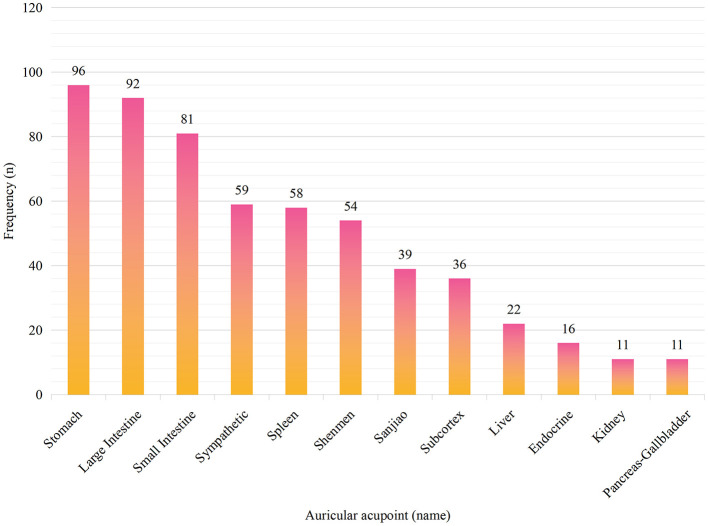
Frequency of auricular acupoints for treating gastrointestinal dysfunction after laparoscopic surgery (frequency ≥ 10).

Among the 118 included papers in this study, there were 28 types of auricular points with a total of 632 occurrences, as seen in Table 1. Among them, there are 12 auricular points with a frequency of occurrence greater than or equal to 10, with a cumulative frequency of 575, which constituted 90.98% of the total percentage, shown in Figure 3, including Stomach, Large Intestine, Small Intestine, Sympathetic, Spleen, Shenmen, Sanjiao, Subcortex, Liver, Endocrine, Kidney, and Pancreas-Gallbladder.

**Table 1:** Co-occurrence counts of top 3 connections in network analysis.

**Strong connection pair**	**Co-occurrence count (*n*)**	**Proportion of total studies (%)**
Large intestine - Stomach	89	75.42
Large intestine - Small intestine	81	68.64
Stomach - Small intestine	77	65.25

### 3.3 Association rule analysis

In association rule analysis, the associations were investigated under the criteria of Support ≥20% and Confidence ≥80%. We generated 48 association rules with lift > 1 which are shown in Tables 2–4, including 11 second-order association rules, 25 third-order association rules, and 12 fourth-order association rules.

**Table 2:** Frequency of auricular acupoints for treating gastrointestinal dysfunction after laparoscopic surgery.

**No**.	**Auricular acupoint**	**Frequency (*n*)**	**Rate (%)**
1	Stomach	96	15.19
2	Large intestine	92	14.56
3	Small intestine	81	12.82
4	Sympathetic	59	9.33
5	Spleen	58	9.18
6	Shenmen	54	8.54
7	Sanjiao	39	6.17
8	Subcortex	36	5.70
9	Liver	22	3.48
10	Endocrine	16	2.53
11	Kidney	11	1.74
12	Pancreas-gallbladder	11	1.74
13	Cardia	7	1.10
14	Lung	7	1.10
15	Duodenum	6	0.95
16	Heart	6	0.95
17	Appendix	5	0.79
18	Brainstem	5	0.79
19	Uterus	5	0.79
20	Occiput	4	0.63
21	Abdomen	2	0.32
22	Distension area	2	0.32
23	Brain	2	0.32
24	Rectum	2	0.32
25	Bladder	1	0.16
26	Middle triangular fossa	1	0.16
27	Adrenal gland	1	0.16
28	Ureter	1	0.16
29	Total	632	100

**Table 3:** Second-order association rules of auricular acupoints for treating gastrointestinal dysfunction after laparoscopic surgery.

**Succedent**	**Antecedent**	**Frequency (*n*)**	**Support**	**Confidence**	**Lift**	**95% CI**
Large intestine	Small intestine	81	68.64	95.06	1.21	92.13–97.99
Large intestine	Sanjiao	39	33.05	94.87	1.21	90.52–99.22
Stomach	Sanjiao	39	33.05	92.30	1.13	86.85–97.75
Large intestine	Spleen	58	49.15	86.20	1.10	80.76–91.64
Stomach	Spleen	58	49.15	86.20	1.05	80.76–91.64
Stomach	Large intestine	92	77.96	85.86	1.05	80.92–90.80
Small intestine	Sanjiao	39	33.05	84.61	1.23	78.53–90.69
Stomach	Small intestine	81	68.64	83.95	1.03	78.68–89.22
Small intestine	Large intestine	92	77.96	83.69	1.21	78.27–89.11
Large intestine	Stomach	96	81.35	82.29	1.05	77.21–87.37
Stomach	Sympathetic	59	50	81.35	1	74.82–87.88

**Table 4:** Third-order association rules of auricular acupoints for treating gastrointestinal dysfunction after laparoscopic surgery.

**Succedent**	**Antecedent**	**Frequency (*n*)**	**Support**	**Confidence**	**Lift**	**95% CI**
Large intestine	Sympathetic + Small intestine	35	29.66	100	1.28	99.12–100.00
Large intestine	Spleen + Small intestine	43	36.44	97.67	1.25	94.53–100.00
Large intestine	Small intestine + Stomach	68	57.62	97.05	1.24	94.38–99.72
Large intestine	Sanjiao + Small intestine	33	27.96	96.96	1.24	93.04–100.00
Large intestine	Subcortex + Small intestine	24	20.33	95.83	1.22	90.08–100.00
Large intestine	Sanjiao + Stomach	36	30.50	94.44	1.21	89.58–99.30
Stomach	Sanjiao + Large intestine	37	31.35	91.89	1.12	86.05–97.73
Stomach	Sanjiao + Small intestine	33	27.96	90.90	1.11	84.52–97.28
Stomach	Sympathetic + Small intestine	35	29.66	88.57	1.08	81.23–95.91
Small intestine	Sanjiao + Large intestine	37	31.35	86.48	1.25	79.15–93.81
Stomach	Spleen + Small intestine	43	36.44	86.04	1.05	78.87–93.21
Stomach	Small intestine + Large intestine	77	65.25	85.71	1.05	80.03–91.39
Stomach	Sympathetic + Large intestine	42	35.59	85.71	1.05	78.27–93.15
Small intestine	Subcortex + Large intestine	27	22.88	85.18	1.24	76.45–93.91
Large intestine	Shenmen + Small intestine	26	22.03	84.61	1.08	75.15–94.07
Small intestine	Large intestine + Stomach	79	66.94	83.54	1.21	77.68–89.40
Small intestine	Sympathetic + Large intestine	42	35.59	83.33	1.21	75.65–91.01
Small intestine	Sanjiao + Stomach	36	30.50	83.33	1.21	75.65–91.01
Stomach	Shenmen + Large intestine	30	25.42	83.33	1.02	74.17–92.49
Stomach	Sympathetic + Spleen	32	27.11	87.5	1.07	79.38–95.62
Stomach	Spleen + Large intestine	50	42.37	86	1.05	79.24–92.76
Large intestine	Spleen + Stomach	50	42.37	86	1.10	79.24–92.76
Small intestine	Spleen + Large intestine	50	42.3	84	1.22	76.68–91.32
Large intestine	Subcortex + Stomach	25	21.18	84	1.07	73.84–94.16
Large intestine	Sympathetic + Spleen	32	27.11	81.25	1.04	71.87–90.63

### 3.4 Clustering analysis

**Figure 4:**
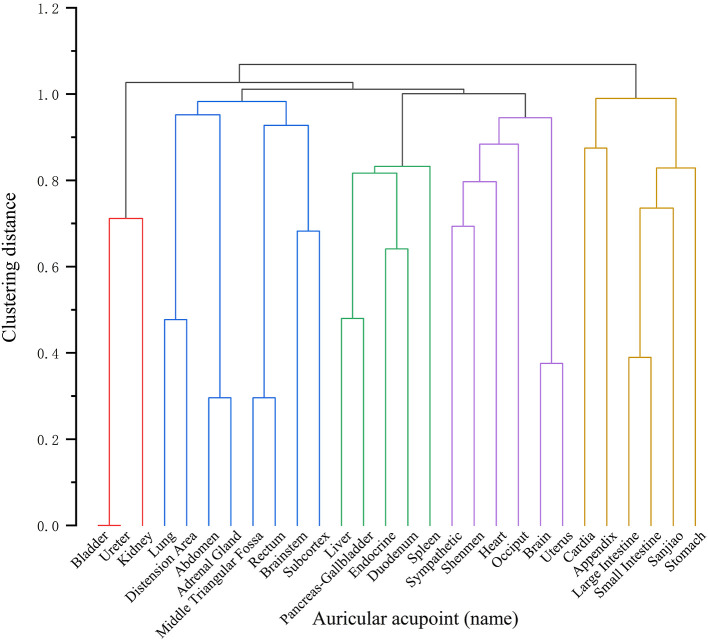
Cluster analysis of auricular acupoints for treating gastrointestinal dysfunction after laparoscopic surgery.

We put 28 high-frequency ear points into OriginPro 2025 for analysis, results of clustering analysis are shown in Figure 4, totally, five clusters are presented, which are: Cluster 1(red): Bladder, Ureter and Kidney. Cluster 2(blue): Lung, Distension area, Abdomen, Adrenal gland, Middle triangular fossa, Rectum, Brainstem, and Subcortex. Cluster 3(green): Liver, Pancreas-Gallbladder, Endocrine, Duodenum, and Spleen. Cluster 4(purple): Sympathetic, Shenmen, Heart, Occiput, Brain, and Uterus. Cluster 5(yellow): Cardia, Appendix, Large intestine, Small intestine, Sanjiao, and Stomach.

### 3.5 Network analysis

**Figure 5:**
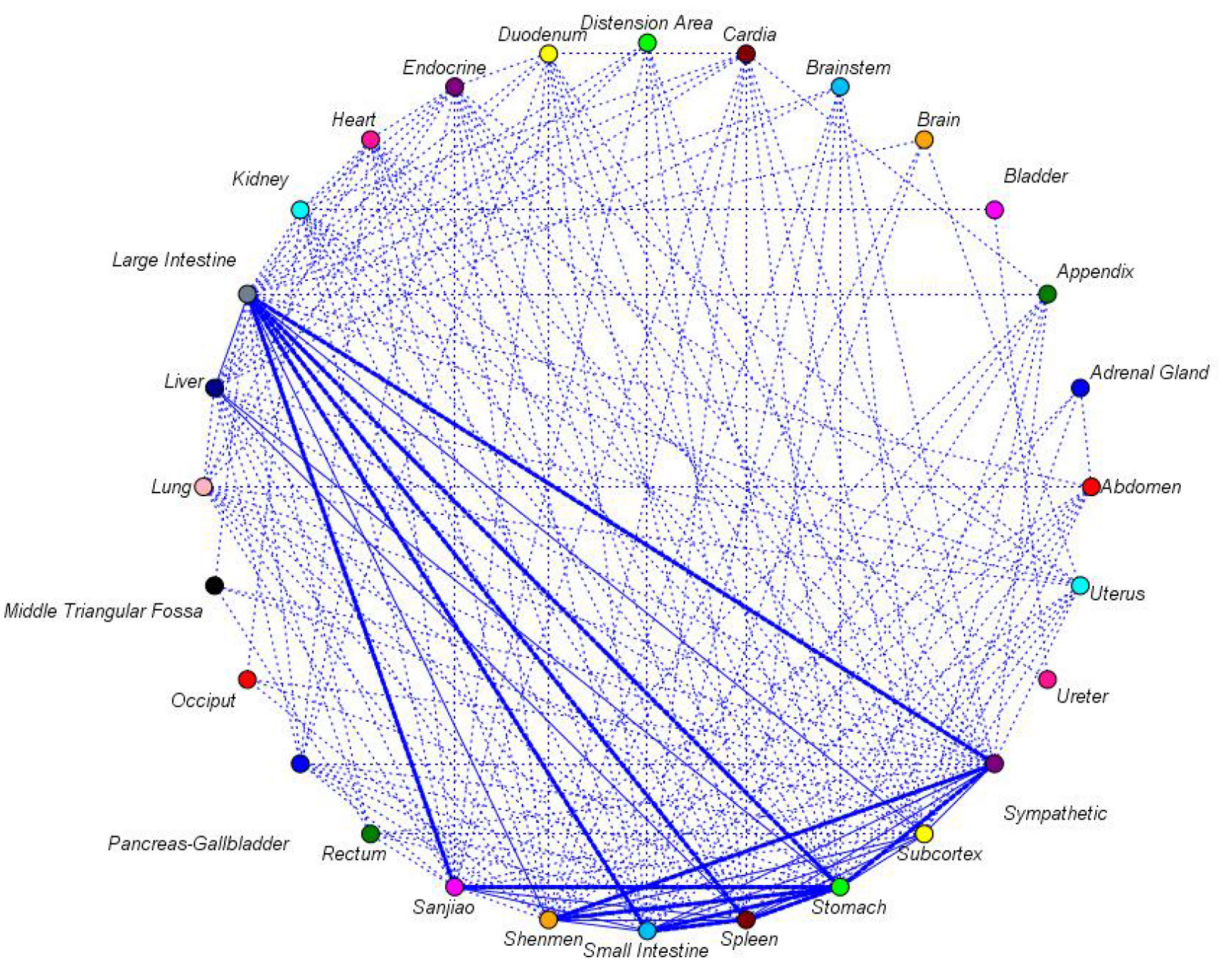
Network analysis of auricular acupoints for treating gastrointestinal dysfunction after laparoscopic surgery.

We set the lower threshold of strong connections at 35 and the upper limit of weak connections at 15. In order to facilitate observation of the network diagram, the display has been set to indicate the strength of the network connections. In network analysis, connections are shown as thick lines for strong connections, solid lines for normal connections, and dotted lines for weak connections. Following a rigorous examination, 12 sets of strong links were discovered; these are detailed below in Figure 5: Large Intestine-Stomach, Large intestine-Small intestine, Stomach-Small intestine, Large Intestine-Spleen, Spleen-Stomach, Sympathetic-Stomach, Spleen-Small intestine, Large Intestine-Sympathetic, Shenmen-Stomach, Large Intestine-Sanjiao, Sanjiao-Stomach, and Sympathetic-Shenmen. Degree centrality reflects the number of direct connections between an acupoint and other acupoints—Stomach, Large Intestine, and Small Intestine ranked top 3, confirming their core status in acupoint combinations. The specific co-occurrence counts of the top 3 strong connections are shown in Table 5. For example, Large Intestine-Stomach co-occurred 89 times (75.42% of total studies), consistent with its high support value (81.35%) in Table 2. These centrality metrics are consistent with frequency analysis results, further verifying the clinical importance of these core acupoints.

**Table 5:** Fourth-order association rules of auricular acupoints for treating gastrointestinal dysfunction after laparoscopic surgery.

**Succedent**	**Antecedent**	**Frequency (*n*)**	**Support**	**Confidence**	**Lift**	**95% CI**
Large intestine	Sympathetic + Small intestine + Stomach	31	26.27	100	1.28	98.95–100.00
Large intestine	Spleen + Small intestine + Stomach	37	31.35	97.29	1.24	93.65–100.00
Large intestine	Sanjiao + Small intestine + Stomach	30	25.42	96.66	1.23	92.03–100.00
Stomach	Sympathetic + Small intestine + Large intestine	35	29.66	88.57	1.08	81.23–95.91
Stomach	Sympathetic + Spleen + Large intestine	26	22.03	88.46	1.08	78.78–98.14
Small intestine	Sympathetic + Large intestine + Stomach	36	30.50	86.11	1.25	78.13–94.09
Stomach	Spleen + Small intestine + Large intestine	42	35.59	85.71	1.05	78.27–93.15
Small intestine	Sanjiao + Large intestine + Stomach	34	28.81	85.29	1.24	76.85–93.73
Small intestine	Sympathetic + Spleen + Large intestine	26	22.03	84.61	1.23	75.15–94.07
Small intestine	Spleen + Large intestine + Stomach	43	36.44	83.72	1.21	76.28–91.16
Large intestine	Sympathetic + Spleen + Stomach	28	23.72	82.14	1.05	72.26–92.02
Stomach	Sanjiao + Small intestine + Large intestine	32	27.11	90.62	1.11	82.98–98.26

## 4 Discussion

Gastrointestinal dysfunction after laparoscopic surgery corresponds to TCM syndromes such as pi-man (fullness due to stagnant qi in the spleen and stomach), abdominal distension, abdominal pain, bi-syndrome (intestinal obstruction, referring to blocked qi and blood in the intestines), (jie-syndrome (intestinal knotting, referring to intestinal qi stagnation and knotting), and constipation. Its pathogenesis is closely linked to surgical trauma-induced qi-blood disharmony and visceral dysfunction. Clinical treatment should target the core pathogenesis of “root deficiency with branch excess” (i.e., underlying deficiency of healthy qi and superficial excess of pathogenic factors) and “obstructed fu-organ qi” (i.e., stagnant qi in the six fu-organs), focusing on three therapeutic directions: fortifying healthy qi to expel pathogenic factors, regulating qi movement, and restoring the normal descending function of the six fu-organs.

Our study demonstrates that the stomach, large intestine, and small intestine acupoints rank among the highest in frequency of use in auricular therapy. This may indicate the holistic concept in Traditional Chinese Medicine (TCM), which is expressed by the axiom, “The ear is the convergence point of all meridians.” Auricular therapy works by stimulating specific areas of the ear to regulate the function of their corresponding internal organs. We emphasize that while our data mining analysis identifies patterns in clinical practice that may demonstrate consistency with predictions from traditional Chinese medicine theory, it does not serve to validate neurobiological mechanisms.

The Stomach (96 cases, 15.19%) functions as a core target within the digestive system. As recorded in Chapter 26 (“Kou Er Wen”/“Questions About the Mouth and Ears”) of Lingshu Jing (Spiritual Pivot): “The ear is connected by the collaterals of the stomach.” The stomach point's frequent use directly corresponds to the pathogenesis of “impaired gastric descent” (wei qi bu jiang) (13) in patients with gastrointestinal dysfunction. Stimulating the stomach point may promote gastrin secretion via the vagus nerve, enhance pepsin activity, accelerate gastric emptying, and improve postoperative digestive function (20, 21). The Large intestine (92 cases, 14.56%) and Small intestine (81 cases, 12.82%) correspond, respectively to the intestinal functions of conveyance and separation of the clear from the turbid. The large intestine governs conveyance (chuan dao), while the small intestine governs transformation (hua wu), consistent with the theory in Suwen Jing “Linglan Mi Dian Lun” (The Plain Questions: The Treatise on the Spiritual Orchid Chamber): “The large intestine plays a pivotal role in the process of elimination and transformation, serving as the primary conduit through which these changes occur.” Modern research indicates that stimulating the large intestine point can regulate Substance P expression in the Enteric Nervous System (ENS), enhance colonic transit motility, alleviate postoperative constipation and abdominal distension, and restore intestinal peristalsis rhythm (22, 23). The Sympathetic (59 cases, 9.33%) has been identified as a critical acupoint for autonomic nervous regulation. Research indicates that by stimulating branches of the great auricular nerve, it can relieve gastrointestinal smooth muscle spasms and alleviate abdominal pain and distension (24, 25). This is indicative and analogous to the regulatory mechanism of the autonomic nervous system on gastrointestinal smooth muscle in modern medicine. The Spleen (58 cases, 9.18%) fortifies the spleen and boosts qi to consolidate the acquired foundation (hou tian zhi ben). The Shenmen (54 cases, 8.54%) tranquilizes the mind and stabilizes the mental state, mitigating the impact of postoperative anxiety on gastrointestinal function. Co-administration of these two points has been evidenced to regulate the limbic-hypothalamic pathway, reduce fluctuations in gastric mucosal blood flow under stressful conditions, and collaboratively enhance gastrointestinal barrier function (26, 27). Experimental studies demonstrate that TCM formulations regulating Sanjiao (39 cases, 6.17%), specifically in the transformation of Qi, as evidenced by the significant enhancement of water metabolism in mice observed in studies involving Wuling San (Five-Ingredient Powder with Poria) (28). Mice treated with Wuling San showed marked improvements in both fecal output volume and water metabolism parameters in the large intestine. The Subcortex (36 cases, 5.70%) has been demonstrated to modulate cerebral cortical function (29), suggesting potential relevance to pain management, emotional regulation, or neurological disorders. As Sanjiao serves as the pathway for primordial qi (yuan qi), while Subcortex corresponds to the projection area of the cerebral cortex, their combined application regulates gastrointestinal motility rhythm through the central-peripheral pathway. This approach is particularly suitable for patients with functional gastrointestinal disorders (FGIDs). The Liver (22 cases, 3.48%) suggests to soothe the liver, promote gallbladder function, and regulate qi movement to enhance digestion (27). The Endocrine (16 cases, 2.53%) has been demonstrated to modulate hormonal balance (e.g., gastrin, insulin) (30). The Kidney (11 cases, 1.74%) may indicate as a crucial point for supporting splenic yang, thereby facilitating the maintenance of metabolic warmth. Similarly, the Pancreas-Gallbladder (11 cases, 1.74%) has been noted to play a regulatory role in glucose metabolism and bile secretion. Collectively, these points establish a “zang-fu simultaneous regulation” framework, covering multifaceted mechanisms of postoperative gastrointestinal functional recovery.

The auricular points that correspond to the Stomach, Large intestine, and Small intestine are located in the cymba conchae and cavum conchae, corresponding to the projection zones of the digestive tract on the auricle. These points form a holographic correspondence with gastrointestinal organs derived from endodermal differentiation during embryonic development. Their innervation is closely associated with the auricular branch of the vagus nerve and the auricular branches of the cervical plexus. Stimulation of these points can directly modulate the myoelectrical activity of gastrointestinal smooth muscle. The Sympathetic is adjacent to the great auricular nerve branches (C2–C3). Its afferent fibers project to the celiac plexus, where they regulate gastrointestinal vasomotor activity and glandular secretion (11). Simultaneously, stimulation can additionally inhibit abnormal discharge frequency in the gastrointestinal smooth muscle of rats and reduce visceral pain sensitivity, known as visceral hyperalgesia (31, 32). The Shenmen point is located in the triangular fossa, which has a direct effect on gastrointestinal regulatory centers, including the celiac plexus and the nucleus tractus solitarius (NTS) in the medulla oblongata, thereby inhibiting over-secretion of acid by gastric parietal cells (12, 33). It further regulates the limbic-hypothalamic-vagus pathway to stabilize gastric mucosal blood flow, exhibiting partial mechanistic overlap with the molecular targets of proton pump inhibitors (PPIs) (34, 35). The association rule analysis of auricular acupoints for treating postoperative gastrointestinal dysfunction following laparoscopic surgery reveals a systematic pattern of acupoint compatibility. This pattern aligns with TCM theories of visceral correlation and meridian conduction. The frequent co-occurrence of acupoints such as Large Intestine, Small Intestine, Stomach, Spleen, and Sanjiao reflects the TCM principle that “the fu-organs function by maintaining unobstructed passage,” underscoring the importance of regulating fu-qi for gastrointestinal recovery.

The second-order association rules indicate that the Large Intestine-Small Intestine pairing occurs most frequently (81 cases; support: 68.64%, confidence: 95.06%; Table 2). In TCM theory, both acupoints correspond to yang fu-organs responsible for food waste transformation and transportation. This synergy is clinically significant: auricular stimulation of the Large Intestine acupoint enhances intestinal peristalsis and reduces postoperative ileus duration (36). Similarly, the high-confidence Large Intestine-Sanjiao combination (39 cases; support: 33.05%, confidence: 94.87%; Table 2) demonstrates Sanjiao's role in regulating qi movement and fluid metabolism. With the passage of water and qi, Sanjiao collaborates with the Large Intestine to eliminate damp-turbidity, which is related to a mechanism critical for resolving postoperative gastrointestinal stasis (14). Third-order association rules reveal two clinically significant combinations. First, the Large Intestine-Sympathetic-Small Intestine triad (35 cases; support: 29.66%, confidence: 100%; Table 3) probably modulates autonomic function to regulate intestinal motility. The Sympathetic acupoint specifically inhibits excessive sympathetic excitability, reducing intestinal smooth muscle spasm (37). Second, the Large Intestine-Spleen-Small Intestine combination (43 cases; support: 36.44%, confidence: 97.67%; Table 3) integrates spleen-strengthening with intestinal regulation, corresponding the core pathogenesis of “spleen deficiency causing intestinal dysfunction.” Clinically, this combination shortens the time to the first postoperative flatus in laparoscopic cases (36). Fourth-order association rules, exemplified by the Large Intestine-Sympathetic-Small Intestine-Stomach combination (31 cases; support: 26.27%; confidence: 100%; Table 4), demonstrate comprehensive regulation of qi-blood-fluid balance. The inclusion of the Stomach acupoint enhances gastric motility modulation, while Sympathetic and Small Intestine collectively maintain intestinal rhythm. This multi-target approach conceptually parallels the TCM formula “Four Miraculous Powder” (Si Miao San), restoring qi flow in the middle jiao. Experimental studies confirm that such multi-acupoint auricular therapy regulates enteric nervous system neurotransmitter acetylcholine to promote gastrointestinal mucosal repair (15). The consistently high lift values (≥1.21) across associations indicate significant synergistic effects, aligning with the TCM principle of “acupoint compatibility for enhanced efficacy.” For instance, the Large Intestine-Small Intestine pair demonstrates a lift of 1.21 (Table 2), predicting 21% higher co-occurrence than expected if used independently. This synergism may arise from their shared vagal innervation, as functional MRI studies confirm that simultaneous stimulation enhances bilateral insular cortex activation (38), which is a key region for gastrointestinal sensory processing.

According to the literature we retrieved, the five clusters cover the main pathological aspects of gastrointestinal dysfunction after laparoscopic surgery, including inflammation, neurological disorders, digestive impairment, and psychological stress, we summarize in a step-by-step therapeutic mechanism: “Regulating the Intestines (Cluster 5) → Calming the Spirit and Regulating Shen (Cluster 4) → Soothing the Liver and Fortifying the Spleen (Cluster 3) → Diffusing Lung Qi and Regulating Qi Movement (Cluster 2) → Regulating Body Fluid Metabolism (Cluster 1).”

Cluster 5 (Yellow: Stomach, Large Intestine, Small Intestine, Sanjiao, etc.), a core regulation of gastrointestinal motility, which is associated with elevated short-chain fatty acids and enhances intestinal mucosal energy metabolism, embodying the TCM principle of “Unblocking Yangming Fu Organs Regulates Intestinal Qi Dynamics.” The stimulation of specific auricular points along the gastrointestinal tract has been shown to directly activate Interstitial Cells of Cajal, elevate motilin levels, and restore gastrointestinal slow-wave rhythm (39, 40). Concurrently, Sanjiao point modulates the thoraco-abdominal pressure gradient, further promotes motilin secretion, optimizes peristaltic biomechanics, and accelerates CO_2_ clearance from the intestinal lumen (41). This mechanism corresponds to the concept of “Unblocking Fu Organs and Regulating Qi.” Clinical case (42) have been shown the efficacy of immediate postoperative application of auricular acupressure combined with moxibustion at Zusanli in facilitating the passage of flatus within 24 h and achieving a complete resolution of abdominal distension. Preventive applications (43, 44) have been shown to demonstrate the efficacy of Cardia point in reducing delayed gastric emptying incidence, and Appendix point in suppressing postoperative intestinal adhesion formation. Cluster 4 (Purple: Sympathetic, Shenmen, Heart, etc.) modulates the neuro-psycho-gastrointestinal axis. The Sympathetic point has been shown to inhibit sympathetic overactivation, suppresses vestibular-vagal nerve excitability, reduces 5-HT release, and restores gastrointestinal smooth muscle rhythm (45). Shenmen activates the nucleus tractus solitarius (NTS) in the medulla oblongata, inhibiting the hypothalamic–pituitary–adrenal (HPA) axis, lowering cortisol levels, and ameliorating stress responses in the “gut-brain axis”(46). In Ma's study (47), Shenmen point combined with moxibustion reduced anxiety scores, may validate the gastrointestinal protective effects of “Calming the Spirit and Regulating Shen.” Research (48) also shows auricular acupressure using sympathetic and stomach reduced the incidence of postoperative nausea and vomiting. Cluster 3 (Green: Liver, Pancreas-Gallbladder, Spleen, etc.) harmonizes liver-spleen and reconstructs digestive function. This approach has been shown to enhance digestive function and promote gastrointestinal motility, which can be related by regulating liver qi, stimulating digestive fluid secretion, and modulating hormone levels. Liver-Pancreas-Gallbladder points have been demonstrated to enhance bile acid concentration and pancreatic lipase activity, which can improve fat digestion (49). Spleen point has been demonstrated to promote the secretion of gastrointestinal hormones, such as cholecystokinin and glucagon-like peptide-1, and to enhance mucosal barrier function, as evidenced by an increase in Lactobacillus (50). As discussed, auricular acupressure at Spleen point demonstrated a significant reduction in postoperative steatorrhea incidence (49), validating the improvement in digestive function through “Fortifying the Spleen and Harmonizing the Stomach.” Researches (51, 52) also demonstrated that auricular acupressure (Spleen, Liver, Pancreas-Gallbladder points) combined with acupoint massage resulted in a significant reduction in bowel sound recovery time, whose mechanism is associated with elevated bile acids and pancreatic lipase. In addition, careful consideration is needed when patients have other underlying conditions at the same time. For diabetic patients (53), the incorporation of Endocrine point has been demonstrated to effectively modulate insulin resistance. However, it is imperative to exercise caution when administering strong stimulation at Liver point in patients with acute hepatitis (54), as this may exacerbate hepatic stress responses. Consequently, it is imperative to take patient medical history into consideration when formulating point protocols to ensure optimal therapeutic outcomes. Cluster 2 (Blue: Lung, Abdominal Distension Area, Adrenal Gland, etc.) impacts Lung-Intestine Qi dynamics coordination and inflammation suppression. This approach has been proven to alleviate postoperative respiratory discomfort and abdominal bloating by regulating respiratory function and attenuating inflammatory responses in surgical patients. The Lung point has been shown to activate the vagus nerve, reduce intra-luminal CO_2_ pressure and enhance intestinal motility (55). The Adrenal Gland point has been evidenced to inhibit inflammatory cytokines such as tumor necrosis factor-α (TNF-α) (56). As detailed in Liu's research (55), stimulation at the Lung point activates via the vagus nerve-intestinal smooth muscle pathway, leading to a reduction in flatus passage time. Cluster 1 (Red: Bladder, Ureter, Kidney) is indirect regulation of fluid metabolism and gastrointestinal function. The stimulation of specific points on the kidneys and bladder has been proven to modulate the secretion of anti-diuretic hormone (ADH) and aldosterone (57). This stimulation has been observed to enhance the contractility of the detrusor muscle in the bladder, promote urination, and reduce intestinal wall edema, also demonstrated to alleviate the mechanical compression of intestinal structures caused by bladder distension, consequently restoring the spatial capacity for gastrointestinal motility. In postoperative urinary retention following gynecological laparoscopy procedures, such as myomectomy, the combination of Uterus point has been demonstrated to enhance efficacy (58). The findings of the integration of auricular acupressure with Zusanli resulted in a significant reduction in the incidence of urinary retention (59).

However, our current study also has some limitations. First, the literature incorporated in this study did not differentiate between auricular stimulation modalities, such as auricular plaster therapy, electro-stimulation, or needle embedding. Efficacy variations attribute to differences in manipulation techniques were not analyzed. This analysis concentrated on the frequency of point occurrence rather than treatment efficacy. Consequently, differing stimulation intensities and modalities may have influenced the reporting frequency of certain point combinations. For instance, in managing postoperative pain, low-frequency auricular electro-acupuncture (24, 40) and vagus nerve stimulation (20, 41, 45) target specific points directly, offering rapid onset and demonstrating greater efficacy in elevating pain thresholds and reducing inflammatory factors like IL-6 and TNF-α. Conversely, traditional Chinese external therapies such as auricular bean pressing (30, 42, 49) provide gentle, safe, and prolonged relief, proving more effective in preventing and treating gastrointestinal adverse reactions like abdominal distension, diarrhea, nausea, and vomiting. The study did not examine outcome-effect relationships, thus precluding inferences about associations between specific point patterns and clinical efficacy. Secondly, the data samples utilized in this analysis may have certain limitations, such as the scope of data sources and individual patient variations. Due to limitations in the original data, a significant proportion of included studies did not report specific surgical procedures or the extent of bowel manipulation, precluding reliable stratification. Consequently, we were only able to differentiate by the site and type of laparoscopic surgery performed. Thirdly, in this study, traditional theories serve solely as the historical and experiential backdrop for clinical practice models in this study, while neurobiological explanations are regarded as hypothetical inferences based on indirect evidence from prior experimental research. It is important to note that these mechanistic interpretations represent hypothesis generation within this study. The underlying mechanisms must be validated through independent biological experiments or outcome-oriented randomized controlled trials, as our data did not directly measure physiological or clinical outcomes.

These factors could affect the generalizability and accuracy of the association rules. Another limitation is the lack of outcome-based analysis. Although the inclusion criterion specified “gastrointestinal function recovery” as the outcome index, correlation between different acupoint combinations and specific outcomes (e.g., time to first flatus, abdominal distension score) did not analyzed. Future studies should adopt a “treatment-outcome” association design to identify the most effective core acupoint clusters. At the same time, studies should integrate modern medical diagnostic modalities and experimental methodologies. Employing advanced technologies like functional Near-Infrared Spectroscopy (fNIRS) (60) and Positron Emission Tomography–Computed Tomography (PET-CT) (61), researchers could dynamically monitor real-time regulatory effects of auricular stimulation on brain-gut axis pivotal nodes (e.g., *insula, amygdala*) to elucidate underlying mechanisms (62). Further investigation into the biological mechanisms of visceral interactions and their crosstalk with the nervous system is essential to establish causal relationships, thereby providing robust support for modernizing TCM theory and enabling precision clinical interventions. Multicenter randomized controlled trials (RCTs) should be conducted to explore disease-specific core acupoint clusters. Additionally, synergistic mechanisms combining auricular therapy with body acupuncture (63) and probiotics (64) warrant investigation to optimize integrated TCM-Western medicine protocols, ultimately enhancing the management of gastrointestinal dysfunction after laparoscopic surgery.

## 5 Conclusion

Our study employs multidimensional data mining (frequency → association → clustering → network) to elucidate the intrinsic logic of auricular acupoint association rules, which is guided by TCM principles of viscera correspondence theory, and meridian connectivity, with scientific validation from modern neuroendocrine and microcirculation research. The identified core formula—comprising the Stomach-Large Intestine-Sanjiao acupoints as the foundational protocol, Sympathetic-Shenmen as the synergistic module, and Spleen as the pivotal modulator for pattern differentiation. A repetitive pattern has been identified in the selection of auricular acupoints for managing gastrointestinal symptoms following laparoscopic surgery, reflecting the application principles summarized in the literature. We will continue to focus on the relevant clinical literature and integrate, further verify and improve the acupoint selection rules of acupoint therapy in the treatment of gastrointestinal dysfunction after laparoscopic surgery, providing a further reference for a future acupoint selection programme of acupoint therapy in the treatment.

## Data Availability

The original contributions presented in the study are included in the article/supplementary material, further inquiries can be directed to the corresponding author.
